# A Rare Case of Shprintzen-Goldberg craniosynostosis syndrome with Hirschsprung disorder: Dental characteristics and its Clinical Management

**DOI:** 10.4317/jced.60930

**Published:** 2023-12-01

**Authors:** Deepak Khandelwal, Shruti-Vinod Kulkarni, Namita Kalra, Rishi Tyagi, Amit Khatri, Padma Yangdol

**Affiliations:** 1 MDS, Assistant Professor, Dentistry (Pedodontics Unit), University College of Medical Sciences and GTB Hospital, Delhi; 2 MDS Student, Dentistry (Pedodontics Unit), University College of Medical Sciences and GTB Hospital, Delhi; 3 MDS Professor, Dentistry (Pedodontics Unit), University College of Medical Sciences and GTB Hospital, Delhi; 4 MDS Senior Resident, Dentistry (Pedodontics Unit), University College of Medical Sciences and GTB Hospital, Delhi

## Abstract

Shprintzen-Goldberg syndrome (SGS) is an autosomal dominant syndrome caused by de novo gene mutations. It is characterized by a number of congenital defects such as craniofacial, skeletal, neurological, and connective tissue abnormalities. It is characterized by craniosynostosis and marfanoid features. To our knowledge, approximately 75 shprintzen-goldberg syndrome cases have been documented since it was first described in 1982. Rare cases of shprintzen-goldberg syndrome have been reported in which the mutated gene was inherited from an unaffected parent through their germline cells i.e., egg or sperm cells. This is a case report of a 6-year-old boy with clinically diagnosed Shprintzen-Goldberg Syndrome with Hirschsprung disease. Patient reported with multiple caries and malpositioned teeth. The treatment initiated with awareness about cariogenic foods, oral hygiene instructions and diet counselling. Subsequently, comprehensive rehabilitation was done.

** Key words:**Dental management, Craniosynostosis, Hirschsprung disorder.

## Introduction

Shprintzen-Goldberg syndrome (SGS) (OMIM #182212) is a very rare congenital disease with incidence of 1 in 1,000,000 which is caused by a heterozygous pathogenic variant in the SKI (Sloan-Kettering Institute) gene (1). It is inherited in an autosomal dominant trait with no gender predilection. It was first introduced by Sugarman and Vogel (1981) and established as an isolated clinical entity in 1982 by Shprintzen and Goldberg.To our knowledge,approximately 75 shprintzen-goldberg syndrome cases have been documented since it was first described (2). It is also known as Marfanoid Craniosynostosis syndrome or Shprintzen-Goldberg craniosynostosis syndrome. Most occurrences are caused by de novo variant individuals without a family history of shprintzen-goldberg syndrome or associated diseases. Rare cases of shprintzen-goldberg syndrome have been reported in which the mutated gene was inherited from an unaffected parent through their germline cells (3) i.e., egg or sperm cells. This kind of mutation, which occurs only in reproductive cells, is referred to as germline mosaicism (4).

Shprintzen-goldberg syndrome comprises of craniofacial anomalies, as well as skeletal, neurological, cardiovascular, and connective-tissue defects (5). The common characteristic features of shprintzen-goldberg syndrome include premature fusion of skull bones resulting in craniosynostosis, long and narrow face, hypertelorism, exophthalmos, broad nose bridge, micrognathia, marfanoid habitus, skeletal malformations, hypotonia, arachnodactyly, omphalocoele and cardiovascular abnormalities. Craniosynostosis usually involving the coronal, sagittal, or lambdoid sutures. Neurodevelopment features include hypotonia, delayed motor and cognitive milestones, mild-to-moderate intellectual disability. Besides this, numerous other features such as aortic root dilatation, Hirschsprung disease, skeletal deformities, mental retardation was also observed (4). Final diagnosis is based on clinical findings and confirmed if mutation is found.

As there is scarcity of case reports about this syndrome in literature, this case report is intended to increase awareness about this rare illness. This case report emphasizes the significance of understanding the craniofacial and oral characteristics for the clinical diagnosis and clinical management of a male child with the extremely unusual Shprintzen-Goldberg’s craniosynostosis syndrome.

This case report aims to present a very rare sporadic case of the syndrome and describe in detail the findings at the maxillofacial region with the comprehensive dental management.

## Case Report

**Figure 1:**
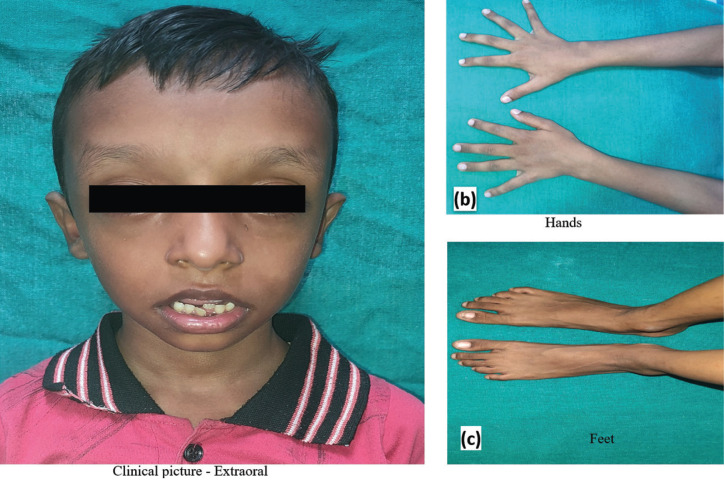
(a) Extraoral picture along with photographs of (b) hands and (c) feet showing arachnodactyly and camptodactyly.

A 6-years-old male child reported to the Department of Pedodontics and Preventive Dentistry at the University College of Medical Sciences and Guru Teg Bahadur Hospital, Delhi, India with the chief complaint of malaligned teeth in lower front teeth region. Patient was a known case of Shprintzen-Goldberg syndrome. Patient was born at 34th gestational weeks with birth weight of 3.3 kg. with no history of consanguineous marriage. Patient had normal antenatal course. Developmental history was normal. Skeletal findings included Pectus carinatum, genu recurvatum, pes planus, thin marfanoid body structure, low subcutaneous fat, camptodactyly in his toes (Fig. 1). He also demonstrated hypermobility of joints.

**Figure 2:**
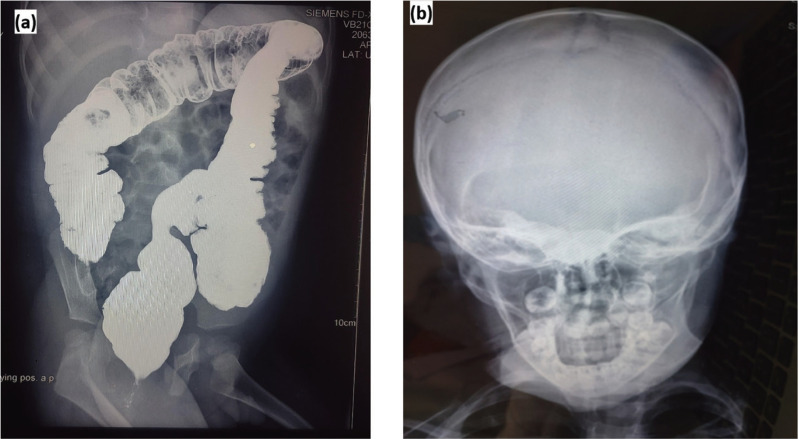
(a) Radiograph during barium enema procedure suggestive of Hirschsprung disease, (b) Skull AP view showing dolichocephaly.

A barium enema was suggestive of Hirschsprung disease (Fig. 2). Absence of ganglion cells and increased acetylcholinesterase staining in nerve fibres were found at rectal biopsy which was done at the age of 2 years which showed findings consistent with the Hirschsprung disease. There was absence of normal RAIR (recto anal inhibitory reflex). It is present when internal anal sphincter relaxation is observed during rectal balloon inflation. In Hirschsprung disease, RAIR is usually absent. No cardiac abnormalities were detected. An Echocardiography revealed normal findings for the age.

Extraoral findings were dolicocephalic pattern, frontal bossing and tall prominent forehead. Exophthalmos, hypertelorism, downward slanting palpebral fissures, ocular proptosis and bilateral blepharoptosis were other key extra oral features. Skull X-rays confirmed dolichocephaly (Fig. 2). Low set ears with prominent helix were observed. Lips were potentially competent. Maxillary hypoplasia with narrow palatal arch and micrognathia resulting in lower incisors crowding was seen. Patient was diagnosed as mouth breather.

**Figure 3:**
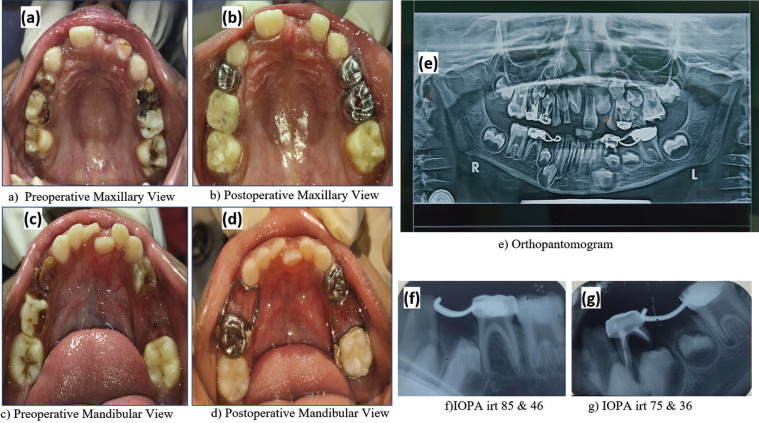
Intraoral pictures along with radiographs (a) Preoperative Maxillary View, (b) Postoperative Maxillary View, (c) Preoperative Mandibular View, (d) Postoperative Mandibular View, (e) Orthopantomogram, f) IOPA irt 85 & 46, g) IOPA irt 75 & 36.

Intraoral examination revealed maxillary hypoplasia, high arched palate, micrognathia, crowding in lower anterior teeth. Patient had mixed dentition with multiple carious and malpositioned teeth. OPG also revealed Taurodontism irt 16,26,36 and 46 Grossly carious 84, chronic irreversible pulpitis with 54,64,55,65 and reversible pulpitis with 16 and 46. 51 was exfoliated and root stumps were present irt 75. Intraoral periapical radiograph revealed absent tooth bud of 45 suggesting hypodontia, (Fig. 3).

The first phase preventive phase initiated with counselling about cariogenic foods, oral hygiene and diet counselling. *Pi*t and fissure sealant was applied irt 26 and 36. In the restorative phase, composite restoration done wrt 16 and 46. Pulpectomy followed by stainless steel crown done with respect to 54, 55 and 74. Stainless steel crown given on 64 for multisurface caries. Extraction was done with respect to 84 and 75. Pulpectomy followed by crown and loop space maintainer done irt 85 to maintain the space for 44. Band and loop space maintainer was fabricated on 36 to preserve space for 35. Strip crown done with 52. Informed consent was obtained from the patient. The patient showed good compliance throughout the procedure. The patient is kept under regular follow-up, (Table 1).

**Table 1:**
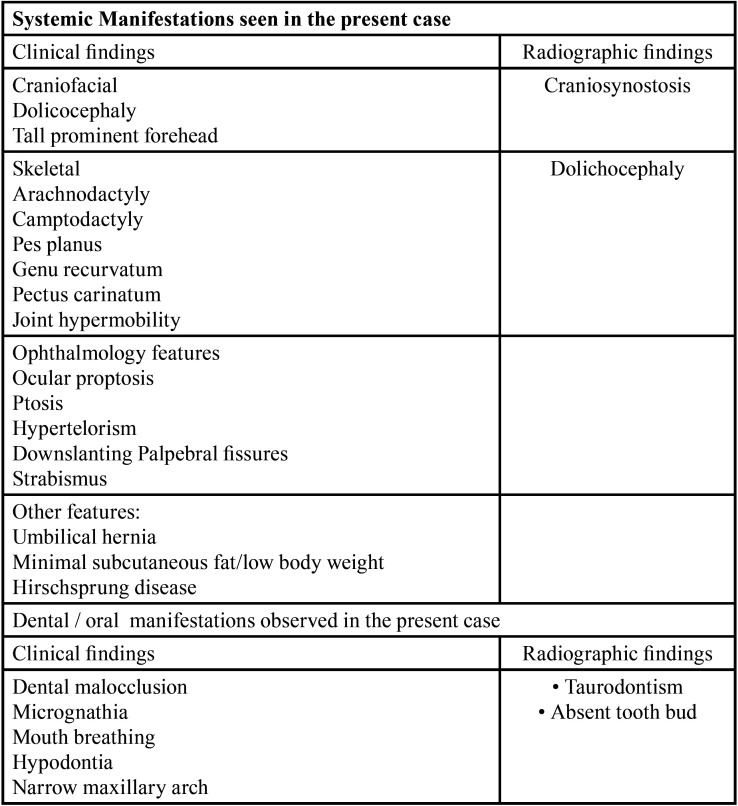
Key characteristics depicting the clinical and radiographic manifestations SGS Syndrome seen in the present case.

## Discussion

Since its discovery in 1982, only around 70 to 80 cases have been reported across the world. Currently, the only known cause of shprintzen-goldberg syndrome is pathogenic mutations in the proto-oncogene SKI, which is a regulator of TGF-activity (4,6). All pathogenic mutations discovered in SKI-positive Shprintzen-Goldberg patients were detected within exon 1, from nucleotide c.63 to c.351 (p.21-p.117) (7). Genetic variations in this gene cause an overactive SMAD-dependent TGF-b signalling pathway. The proto-oncoprotein SKI typically suppresses SMAD proteins by inhibiting them from entering the nucleus to transcribe the TGF-b gene. Cell proliferation, growth, and programmed cell death are all regulated by the TGF-b pathway. Its dysregulation causes numerous cardiovascular and connective tissue defects seen in shprintzen-goldberg syndrome (6). The fibrillin 1 (FBN1) gene, which additionally codes for a TGF-b regulatory protein, is less frequently mutated in shprintzen-goldberg syndrome patients. Excess activity can be caused by mutations in other proteins on this pathway, resulting in phenotypic manifestations similar to those seen in Marfan and Loeys-Dietz syndromes (8).

In differential diagnosis of Shprintzen Goldberg syndrome, Loeys-Dietz syndrome and Marfan syndrome, frontometaphyseal dysplasia, the Melnick–Needles syndrome (MNS), the Idaho syndrome-II can be considered (2). Other syndromic conditions such as Stickler syndrome and otopalatodigital syndrome show some degree of overlap but craniofacial anomalies specific to shprintzen-goldberg syndrome aid to distinguish it from these syndromes. Previously, diagnosis of shprintzen-goldberg syndrome was challenging due to the substantial overlap with Marfan and Loeys-Dietz syndrome as well as diagnosis was only relied on recognizing characteristic features. In recent years, it is evident that Shprintzen-Goldberg syndrome has a unique manifestation as the underlying genetic basis for this syndrome has been revealed. Shprintzen-goldberg syndrome has distinct craniosynostosis and mild to moderate intellectual disability which distinguishes it from Marfan and Loeys Dietz syndrome. Some radiographic abnormalities are more frequently seen in shprintzen-goldberg syndrome than in LDS and MFS such as C1/C2 anomalies, 13 pairs of ribs, square shaped vertebra, Chiari 1 malformation (9). Craniofacial phenotype is very common in shprintzen-goldberg syndrome patients. All patients with mutation in SKI gene have abnormal dolichocephaly or scaphocephaly, hypertelorism, down slanting (anti-mongoloid) palpebral fissures and proptosis along with this micrognathia or retrognathia is frequently seen. This is in accordance with the previously published clinical analysis of Robinson *et al*. (5) [2005] who reviewed 14 new patients and 23 previously documented individuals, and Greally *et al*. (10) [1998] who reviewed four new patients and 11 previously reported individuals, including a clinical re-evaluation of one original patient described by Shprintzen and Goldberg in 1982. Greally *et al*. (10) [1998] stated that almost every patient had dolicochephaly, hypertelorism with downslanting palpebral fissures, proptosis, midface hypoplasia and micrognathia. Robinson *et al*. [2005] reported that more than two-thirds of those with a clinical diagnosis of Shprintzen-Goldberg syndrome described having this characteristic facial appearance (5).

The dolichocephalic head and facial dysmorphism are caused by the premature fusion of the cranial sutures, which impairs the normal growth of the cranial and facial bones (11). These features seen in the present case is in accordance with the previous case reports published all over the world (12,13). Marfanoid habitus is also evident in the present case.

Intellectual disability is one of the characteristic features of Shprintzen-Goldberg syndrome. Contrary to this, in the present case intellectual disability was absent. There have been numerous patients with normal intelligence who exhibited craniosynostosis, marfanoid body habitus, and craniofacial traits suggestive of Shprintzen-Goldberg syndrome. [Furlong *et al*., 1987; Lacombe and Battin, 1993; Megarbane and Hokayem, 1998; Carmignac *et al*., 2012] (7).

In the present case dental characteristics associated with SGCS such as maxillary hypoplasia, dental malocclusion, micrognathia, high-arched palate,hypodontia and mouth breathing were observed.

In contrast to the previously reported cases in literature, there was no abnormality associated with the palate such as cleft palate, prominent ridges, bifid uvula or absent uvula (11,13) in the present case. Congenital absence of permanent toothbud of mandibular second premolar in the present case was consistent with the case of 8 years female patient reported by Topouzelis *et al*. (13) in which panoramic radiograph revealed absence of multiple congenital permanent teeth particularly lower lateral incisors and second maxillary premolars.

Pavone *et al*. (14) reported a case of a 16 years male patient with 12 years follow-up showing clinical features characteristic of shprintzen-goldberg syndrome in addition to many dental findings such as hypodontia, impacted teeth and abnormalities associated with root anatomy and pulp canal shape. Consistent with this case, in the present case, intraoral periapical radiograph shows absent tooth bud wrt 45.

O’Dougherty *et al*. (1) described a case report of a patient with midface hypoplasia causing breathing difficulties and obstructive sleep apnoea due to severe airway obstruction. Surgery was done to treat midface hypoplasia. The severity of these symptoms should be considered against the potential risks of undergoing this type of surgery (1).

It is imperative to prevent dental diseases in shprintzen-goldberg syndrome patients and manage dental manifestations of shprintzen-goldberg syndrome , thereby maintaining good oral health, and enhancing patients’ quality of life. For this purpose, dental approach to patients with shprintzen-goldberg syndrome should integrate a multidisciplinary dental team involving paediatric dentistry, orthodontics and oral and maxillofacial surgeons. According to AAPD (American Academy of Paediatric Dentistry) guidelines on caries risk assessment , children with special healthcare needs are at a moderate caries risk category. Patient’s quality of life can be improved simultaneously lowering their risk of caries by developing a patient-specific dental care plan that includes hygiene guidelines for caretakers and referring the patient to other specialists, with a multidisciplinary management approach (15). General dentist or paediatric dentist should be able to recognize external morphologic phenotype characteristic to shprintzen-goldberg syndrome. It is critical for determination of etiologic factors, treatment planning ,prognosis and genetic counselling (2).

## Conclusions

Patients with SGCS should receive the good dental treatment from young age. Parents should be made aware about child’s special oral hygiene needs, to reduce the risk of dental problems and in order to avoid sufferings due to pain and unnecessary invasive treatment. This case report emphasizes the significance of understanding the craniofacial and oral characteristics for the clinical diagnosis and clinical management of a male child with the extremely unusual Shprintzen-Goldberg’s craniosynostosis syndrome.

## References

[1] O'Dougherty GR, Fulkerson DH, Kern M, Haldar K, Calhoun B (2019). Complications of Insufficient Dura and Blood Loss During Surgical Intervention in Shprintzen-Goldberg Syndrome: A Case Report. Am J Case Rep.

[2] Vieira DM, Silva FG, Diniz MB, Ferreira MCD, Santos MT, Guaré RO (2022). Shprintzen-goldberg craniosynostosis: craniofacial and oral characteristics, diagnosis, and clinical management of a very rare syndrome. RGO, Rev Gaúch Odontol [Internet].

[3] Choi JH, Li R, Gannaway R, Causey TN, Harrison A, Couser NL (2020). Eye Manifestations of Shprintzen-Goldberg Craniosynostosis Syndrome: A Case Report and Systematic Review. Case Rep Genet.

[4] Yadav S, Rawal G (2016). Shprintzen-Goldberg syndrome: a rare disorder. Pan Afr Med J.

[5] Robinson PN, Neumann LM, Demuth S, Enders H, Jung U, König R (2005). Shprintzen-Goldberg syndrome: fourteen new patients and a clinical analysis. Am J Med Genet A.

[6] Doyle AJ, Doyle JJ, Bessling SL, Maragh S, Lindsay ME, Schepers D et al (2012). Mutations in the TGF-β repressor SKI cause Shprintzen-Goldberg syndrome with aortic aneurysm. Nat genet.

[7] Au PY, Racher HE, Graham JM, Kramer N, Lowry RB, Parboosingh JS (2014). De novo exon 1 missense mutations of SKI and Shprintzen-Goldberg syndrome: two new cases and a clinical review. Am J Med Genet A.

[8] Wheeler JB, Ikonomidis JS, Jones JA (2015). Connective tissue disorders and cardiovascular complications: The indomitable role of transforming growth factor-beta signaling. Adv Exp Med Biol.

[9] Yalcintepe S, Yüreğir OO, Bozdoğan ST, Aslan H (2015). Shprintzen-Goldberg Syndrome: Case Report. Meandros Med Dent J.

[10] Greally MT, Carey JC, Milewicz DM, Hudgins L, Goldberg RB, Shprintzen RJ (1998). Shprintzen-Goldberg Syndrome: A Clinical Analysis. Am J Med Genet.

[11] Raseena KT, Kumar A, Jeeva PP, Ramesh R (2020). Shprintzen-Goldberg syndrome (SGS): an extremely rare disorder. IOSR-JDMS.

[12] Waterlow JC (1972). Classification and definition of protein-calorie malnutrition. British medical journal.

[13] Topouzelis N, Markovitsi E, Antoniades K (2003). Shprintzen-Goldberg syndrome: case report. Cleft Palate Craniofac J.

[14] Pavone V, Leonardi R, Sorge G, Pavone P, Pratico A, Sessa G (2012). A patient with Shprintzen-Goldberg syndrome. Clinical follow-up for twelve years. J Pediatr Sci.

[15] American Academy of Pediatric Dentistry (2017). Reference manual. Guideline on Caries-risk Assessment and Management for Infants, Children, and Adolescents. Pediatr Dent.

